# Evaluation and application analysis of animal models of PIPNP based on data mining

**DOI:** 10.1515/biol-2025-1122

**Published:** 2025-07-08

**Authors:** Jun Yu, Shengbo Jin, Haozhe Piao, Mingzhu Li

**Affiliations:** Liaoning University of Traditional Chinese Medicine, Shenyang, Liaoning, 110847, P.R. China; College of Acupuncture and Massage of Liaoning University of Traditional Chinese Medicine, Shenyang, Liaoning, 110847, P.R. China; Department of Neurosurgery, Cancer Hospital of China Medical University, Liaoning Cancer Hospital & Institute, Shenyang, Liaoning, 110042, P.R. China; Department of Integrated Traditional Chinese and Western Medicine Medical Oncology, Cancer Hospital of China Medical University, Liaoning Cancer Hospital & Institute, Shenyang, Liaoning, 110042, P.R. China

**Keywords:** paclitaxel, peripheral neuropathic pain, animal models, data mining

## Abstract

The aim of this study is to systematically retrieve, synthesize, and assess methodologies employed in the development of paclitaxel-induced peripheral neuropathic pain (PIPNP) animal models, with the objective of optimizing protocols for model construction and evaluation. A structured search strategy was implemented using the terms “Paclitaxel-induced peripheral neuropathic pain” OR “Chemotherapy-induced peripheral neuropathic pain” AND “animal models” OR “rats” OR “mice” OR “experimental animals” across the China National Knowledge Infrastructure, Wanfang, and VIP databases. Identical search criteria were applied to the PubMed and Web of Science platforms. The literature search covered publications from database inception through December 31, 2024. A total of 128 articles were reviewed, including 18 in Chinese and 110 in English. All studies employed rodent models, including 16 strains and including both sexes, with ages ranging from 5 weeks to 10 months. In rats, 28 distinct modeling protocols were identified: 4 involving continuous paclitaxel injections, 23 employing alternate-day injections, and 1 using a single administration. For mice, 21 methods were recorded, comprising 6 continuous, 13 alternate-day, and 2 single-injection protocols. Upon successful model induction, six behavioral pain alterations were consistently documented, evaluated using 16 different assessment techniques. Current PIPNP models predominantly utilize 6- to 8-week-old male Sprague-Dawley (SD) rats, with paclitaxel administered at 2 mg/kg on either days 0, 2, 4, and 6 or days 1, 3, 5, and 7. Based on data mining and comparative evaluation of modeling approaches, the use of 6- to 8-week-old male SD rats or 8- to 10-week-old male C57BL/6J mice with paclitaxel administered at 2 mg/kg on days 1, 3, 5, and 7 is recommended for establishing PIPNP models. The translational alignment between PIPNP models and clinical phenotypes remains insufficient and requires further refinement.

## 1 Introduction

Malignant tumors remain the leading cause of death worldwide, significantly impeding efforts to enhance global life expectancy [1]. Data from the International Agency for Research on Cancer indicate that approximately 20 million new cancer cases were diagnosed globally in 2022, accompanied by 9.7 million cancer-related deaths, both figures inclusive of non-melanoma skin cancers. By 2050, the global cancer burden is projected to escalate to 35 million new cases annually [2]. This trajectory firmly establishes malignant tumors as the dominant global health threat [3]. Paclitaxel, a broad-spectrum chemotherapeutic primarily extracted from medicinal plants such as the yew and Taxus chinensis [4], continues to serve as a standard first-line treatment for multiple malignancies, including breast, lung, and ovarian cancers [5]. Despite its efficacy, paclitaxel therapy is frequently compromised by severe side effects, particularly paclitaxel-induced peripheral neuropathic pain (PIPNP), which affects up to 87% of recipients [6]. Characterized by persistent sensory disturbances – numbness, tingling, and pain in the extremities – PIPNP may also impair motor function, often persisting for extended periods and leading to significant treatment interruptions and deterioration in patients’ quality of life [7]. The pathogenesis of PIPNP remains poorly understood, hindering the development of effective preventive and therapeutic strategies. Current interventions for chronic PIPNP are limited, with duloxetine being the sole pharmacological agent conditionally endorsed by the American Society of Clinical Oncology guidelines, primarily for cases associated with platinum compounds rather than taxanes [8]. Despite growing interest in mitigating PIPNP, its underlying mechanisms and preventive measures continue to present substantial research challenges. Progress in this area relies on the establishment of a robust and reproducible animal model; however, agreement is lacking regarding optimal species selection, modeling protocols, and assessment methodologies. This study presents a comprehensive analysis of preclinical models to inform the development of a standardized framework, emphasizing critical variables including animal species, induction methods, pain-related behaviors, evaluation strategies, and relevant biomarkers.

## 2 Materials and methods

### 2.1 Data sources and search strategy

A comprehensive search was performed across China National Knowledge Infrastructure, Wanfang, and VIP databases (from inception to December 31, 2024) using the following terms: (“Paclitaxel-induced peripheral neuropathic pain” OR “Chemotherapy-induced peripheral neuropathic pain”) AND (“animal models” OR “mice” OR “rats” OR “experimental animals”). The same strategy was applied to PubMed and Web of Science for the same period. Only documented queries were retained, with detailed search terms presented in Table 1.

**Table 1:** List of main search formats

Serial number	Search formats
1	Themes = “Paclitaxel-induced peripheral neuropathic pain” + “animal models”
2	Themes = “Paclitaxel-induced peripheral neuropathic pain” + “rats”
3	Themes = “Paclitaxel-induced peripheral neuropathic pain” + “mice”
4	Themes = “Paclitaxel-induced peripheral neuropathic pain” + “experimental animals”
5	Themes = “Chemotherapy-induced peripheral neuropathic pain” + “animal models”
6	Themes = “Chemotherapeutic alcohol-induced peripheral neuropathic pain” + “rats”
7	Themes = “Chemotherapy-induced peripheral neuropathic pain” + “mice”
8	Themes = “Chemotherapy-induced peripheral neuropathic pain” + “experimental animals”

### 2.2 Literature screening and selection criteria

A total of 467 records were initially retrieved. Following the removal of duplicates from both Chinese and English databases, the remaining entries underwent further screening to enhance literature quality. Conference abstracts, monographs, reviews, and records with incomplete data were excluded. Ultimately, 128 studies involving animal experiments on PIPNP were selected based on predefined inclusion criteria.

### 2.3 Statistical analysis

The experimental animal specifications adhered to the guidelines outlined in Experimental Animals and Animal Experimental Techniques. Data concerning animal selection (species, gender, and age), modeling approach, paclitaxel administration method, pain behavior during model induction, detection protocols, solvent choice for paclitaxel, safety profile, application contexts, and relevant experimental indicators were systematically recorded in Microsoft Excel 2021 to construct the PIPNP animal model database. Subsequent data processing – including summarization, cleaning, and analysis – was conducted using the same software. Due to missing information in some cases, the sum of individual data points in the statistical results may not add up to the total, and the final statistical results shall prevail.

## 3 Results

### 3.1 Experimental animal attributes

The 128 included documents contained 16 rodent species and strains, comprising two rat species – Sprague-Dawley (SD) and Wistar rats – with SD rats used more frequently (68 instances, 51.91%), and 14 mouse strains, including C57BL, C57BL/6, C57BL/NRj, C57BL/c, ICR, CD1, Swiss albino, ddY, CF-1, NMRI, s1-KO CD-1, and σ1R-KO CD-1. C57BL/6, C57BL/6J, and CD1 mice appeared most frequently (8 instances, 6.11%). Three studies lacked specific strain identification, referring only to rats or mice, while another three employed dual mouse strains for modeling. In total, rodent species were cited 128 times, as presented in Table 2. Male animals were predominantly used (96 instances, 75.00%), although eight studies omitted gender specification. Gender-related data are detailed in Table 3. Most subjects were aged 8–12 weeks (23 instances, 17.97%), followed by those aged 6–8 weeks (17 instances, 13.28%), and 7–9 weeks (5 instances, 3.91%). Eighteen studies indicated the use of adult mice without further age clarification, while 53 provided no age information. Age-related data are summarized in Table 4.

**Table 2:** PIPNP model – animal species and their usage frequency

Species	Frequency (instances)	Frequency/total (%)	Refs.
SD rats	68	51.91	[9,10,11,12,13,14,15,16,17,18,19,20,21,22, 23,24,25,26,27,28,29,30,31,32,33,34, 35,36,37,38,39,40,41,42,43, 44,45,46,47,48,49,50,51,52,53,54,55,56,57,58, 59,60,61,62,63,64,65,66,67, 68,69,70,71,72,73,74,75,76]
Wistar rats	13	9.92	[77,78,79,80,81,82,83, 84,85,86,87,88,89]
C57BL mice	2	1.53	[90,91]
C57 BL/6 mice	8	6.11	[92,93,94, 95,96,97,98,99]
C57 BL/6J mice	8	6.11	[100,101, 102,103,104,105,106,107]
C57BL/6N mice	2	1.53	[108,109]
C57BL/NRj mice	1	0.76	[110]
C57BL/c mice	6	4.58	[111,112,113,114,115,116]
ICR mice	6	4.58	[26,117,118,119,120,121]
CD1 mice	8	6.11	[122,123,124,125,126,127,128,129]
Swiss albino	1	0.76	[130]
ddY mice	1	0.76	[131]
CF-1 mice	1	0.76	[132]
NMRI mice	1	0.76	[133]
s1-KO CD-1 mice	1	0.76	[88]
σ1R-KO CD-1 mice	1	0.76	[89]
Not mentioned	3	2.29	[134,135,136]
Total	131	100	

**Table 3:** PIPNP model – animal gender and their usage frequency

Gender	Frequency (instances)	Frequency/total (%)	Refs.
Both male and female	12	9.38	[20,30,36,85,97,104,107,109,110,121,127,128]
Female	12	9.38	[38,69,72,80,88,89,101,111,113,114,117,124]
Male	96	75.00	[9,10,11,12,13,14,15,16,17,18, 19,21,22,23,24,25,26,27,29,31,32,33,34,35,37,39,40,41, 42,43,44,45,46,47,48,49,50,51,52,53,54,55,56, 57,58,59,60,61,62,63,64,65,66,67,68, 69,70,71,73,74,75,76,77,78,79,81,82,84,86,87,91,92,93,95,96,98,100,102,103,105,106,108,112,115,117,118,119,120,122,123,126,129,130,131,132,134]
Not mentioned	8	6.25	[28,83,90,94,125,133,135,136]
Total	128	100	

**Table 4:** PIPNP model – animal age and their usage frequency

Age	Frequency (instances)	Frequency/total (%)	Refs.
10 months	1	0.78	[130]
3–4 months	2	1.56	[40,123]
12–14 weeks	2	1.56	[100,103]
10 weeks	2	1.56	[28,104]
8–12 weeks	23	17.97	[59,61,65,67,71,78,85,90,91,93,97, 99,107,108,110,111,113,114,115,116,120,121,129]
6–12 weeks	1	0.78	[109]
7–9 weeks	5	3.91	[9,51,87,95,122]
6–8 weeks	17	13.28	[17,23,25,33,44,58,73,81,86,92,94,96, 98,102,106,126,127]
5–8 weeks	1	0.78	[43]
5–6 weeks	3	2.34	[24,101,117]
Adult	18	14.06	[10,11,14,18,21,26,30,32,34, 35,39,45,53,57,76,82,84,132]
Not mentioned	53	41.41	[12,13,15,16,19,20,22,27,29,31, 36,37,38,41,42,46,47,48,49,50,52,54, 55,56,60,62,63,64,66,68,69,70,72,74,75,77,79,80,83,88, 89,105,112,118,119,124,125,128,131,133,134,135,136]
Total	128	100	

### 3.2 Paclitaxel solvent

Of the 128 documents reviewed, 18 distinct methods for paclitaxel solvent preparation were identified. The most commonly reported combinations included Cremophor EL/ethanol (1:1) with 0.9% saline (38 instances, 29.69%) and 0.9% saline alone (24 instances, 18.75%). In 23 documents, the solvent preparation method was either omitted or not specified. A comprehensive summary is presented in Table 5.

**Table 5:** PIPNP model – paclitaxel solvent and their usage frequency

Paclitaxel solvent	Frequency (instances)	Frequency/total (%)	Refs.
0.9% saline solution	24	18.75	[11,14,19,26,41,47,49,52,55,56, 60,62,65,67,69, 71,75,112,120,124,126,127,131]
Cremophor EL/ethanol (1:1)	13	10.16	[9,13,18,34,35,42,73,84,87,89,121,129,132]
Cremophor EL/ethanol (1:1) + 0.9% saline solution	38	29.69	[15,27,29,31,32,36,37,40,43,44,46, 50,51,58,64, 70,72,85,88,92,93,99,100,103,104,105, 108,109,110,111,113,114,115,116,117,122,135,136]
Cremophor EL/ethanol (1:1) + PBS solution	8	6.25	[81,91,94,96,98,102,106,128]
Cremophor EL/ethanol (1:1) + PBS solution + DMSO	1	0.78	[107]
5% DMSO + 40% PEG 300 + 5%Tween 80 + ddH2O	1	0.78	[123]
40% DMSO	2	1.56	[21,118]
10% DMSO	1	0.78	[39]
10% DMSO + 20%PEG300 + 10％Tween 80 + 0.9% saline solution	1	0.78	[53]
10％ DMSO + 40％PEG300 + 5%Tween 80 + 0.9% saline solution	1	0.78	[17]
4% DMSO + 4%Tween80 + 0.9% saline solution	1	0.78	[54]
2%DMSO + 0.9% saline solution	1	0.78	[78]
DMSO/Tween80 (1:1) + 0.9%saline solution	1	0.78	[16]
Cremophor EL	2	1.56	[63,76]
DMSO (concentration not mentioned) + 0.9% saline solution	6	4.69	[10,23,24,48,83,95]
PBS solution	1	0.78	[22]
99.9% HPLC-grade methanol + 0.9% saline solution	1	0.78	[82]
Cremophor EL + 10% saline solution	1	0.78	[125]
5% DMSO	1	0.78	[119]
Not mentioned	23	17.97	[12,20,25,28,30,33,38,45,57,59,61,66, 74,77,79, 80,86,90,97,101,130,133,134]
Total	128	100	

### 3.3 Paclitaxel injection methods

Of the 128 studies reviewed, model induction was predominantly achieved via intraperitoneal injection, accounting for 127 instances (99.22%) in rats or mice. In contrast, intravenous administration was rarely applied, appearing in only a single instance (0.78%), as presented in Table 6.

**Table 6:** PIPNP model – paclitaxel injection methods and their usage frequency

Injection methods	Frequency (instances)	Frequency/total (%)	Refs.
Intraperitoneal injection	127	99.22	[9,10,11,12,13,14,15,16,17,18,19,20,21,22, 23,24,25,26,27,28,29,30,31,32,33,34, 35,36,37,38,39,40,41,42,43,44,45,46, 47,48,49,50,51,52,53,54,55,56,57,58,59,60,61, 62,63,64,65,66,67,68,69,70,71,73,74, 75,76,77,78,79,80,81,82,83,84,85,86,87,88,89, 90,91,92,93,94,95,96,97,98,99,100,101,102,103,104, 105,106,107,108,109,110,111,112,113,114,115,116,117,118,119,120,121,122,123,124,125,126,127,128, 129,130,131,132,133,134,135,136]
Intravenous injection	1	0.78	[72]
Total	128	100	

### 3.4 Modeling protocols

In patients with malignant tumors receiving paclitaxel chemotherapy, acute neuropathic symptoms typically emerge within 24–72 h following a single dose of 135 or 175 mg/m^2^ (equivalent to 3.6 and 4.7 mg/kg, respectively), in the absence of detectable changes in joint or musculoskeletal structures. These transient symptoms usually resolve within 1 week post-infusion. When paclitaxel is administered intravenously every 3 weeks over 3–24 h, with cumulative doses ranging from 1,400 to 1,500 mg/m^2^ (37.8–40.5 mg/kg), patients frequently exhibit chronic manifestations, including sensory deficits, paresthesia, or functional impairments impacting daily life. Consequently, current rat and mouse PIPNP models predominantly replicate chronic pain phenotypes, while validated models of the acute phase remain lacking [137,138]. Literature mining identified 28 rat model induction protocols, comprising 4 continuous dosing strategies, 23 alternating-day regimens, and 1 single-dose protocol. The most prevalent schedules involved 2 mg/kg injections on days 0, 2, 4, and 6 (17 instances, 20.48%) and on days 1, 3, 5, and 7 (24 instances, 28.92%), as summarized in Table 7. For mouse models, 21 distinct approaches were documented, including 6 continuous dosing regimens, 13 alternating-day protocols, and 2 single-dose methods. The most frequently reported included 2 mg/kg on days 1–5 (12 instances, 24.49%) and on days 0, 2, 4, and 6 (9 instances, 18.37%), with details outlined in Table 8. Among the 128 studies reviewed, 80 employed rat-based modeling strategies, 45 utilized mouse-based approaches (including 1 study applying both), and 3 incorporated both species for model development.

**Table 7:** PIPNP rat model – modeling protocols and their usage frequency

Modeling protocols	Specific protocols	Frequency (instances)	Frequency/total (%)	Refs.
Alternate-day injection protocols	2 mg/kg, injected once every other day, for a total of four injections	4	4.82	[28,36,51,134]
	2 mg/kg, injected every 5 days, for a total of five injections	1	1.20	[84]
	2 mg/kg, injected every 4 days for a total of four injections	1	1.20	[76]
	3 mg/kg, injected once every other day, for a total of four injections	1	1.20	[70]
	6 mg/kg, injected once a week for 4 weeks	1	1.20	[74]
	16 mg/kg, injected once a week for 5 weeks	1	1.20	[75]
	1 mg/kg, injected on days 1, 3, 5, and 7	3	3.61	[21,39,63]
	1 mg/kg, injected on days 0, 2, 4, and 6	2	2.41	[41,83]
	2.47 mg/kg, injected on days 1, 3, 5, and 7	3	3.61	[60,65,71]
	2 mg/kg, injected on days 0, 2, 4, and 6	17	20.48	[11,15,16,17,19,20,22,23,30,42, 45,53,54,64,80,81,87]
	2 mg/kg, injected on days 1, 3, 5, and 7	24	28.92	[13,14,18,24,26,29,31,32,34, 35,43,45,48,50,58,59,61,62, 66,67,68,78,79,136]
	2 mg/kg, injected on days 1, 3, 5, 7, and 9	1	1.20	[10]
	3, 6 mg/kg, injected on days 1, 2, 8, 9, 15, 16, 22, and 23	1	1.20	[9]
	4 mg/kg, injected on days 0, 2, 4, and 6	1	1.20	[56]
	4 mg/kg, injected on days 1, 3, 5, and 7	2	2.41	[12,38]
	4 mg/kg, injected on days 1, 4, 8, and 11	1	1.20	[73]
	4, 6 mg/kg, injected on days 1, 8, and 15	1	1.20	[57]
	5 mg/kg, injected on days 0, 2, 4, and 6	1	1.20	[69]
	5 mg/kg, injected on days 0, 7, 14, and 21	1	1.20	[27]
	8 mg/kg, injected on days 1, 4, and 7	5	6.02	[40,47,49,52,55]
	8 mg/kg, injected on days 0, 3, and 6	1	1.20	[85]
	8 mg/kg, injected on days1, 3, 5, 7, and 9	1	1.20	[25]
	9 mg/kg, injected on days 0, 2, 4, and 6	1	1.20	[77]
Continuous injection protocols	2 mg/kg, injection on days 1–4	1	1.20	[44]
	2 mg/kg, injection on days 1–5	4	4.82	[33,86,88,89]
	2 mg/kg, injection on days 0–6	1	1.20	[82]
	3–7 mg/kg, injection on days 1–5	1	1.20	[72]
Single administration protocols	5, 10 mg/kg, injected once	1	1.20	[37]
	Total	83	100	

**Table 8:** PIPNP mouse model – modeling protocols and their usage frequency

Modeling protocols	Specific protocol	Frequency (instances)	Frequency/total (%)	Refs.
Alternate-day injection protocols	4 mg/kg, injected once every other day, for a total of four injections	1	2.04	[129]
	4 mg/kg, injected once every other day, for a total of six injections	1	2.04	[93]
	4 mg/kg, injected once every other day, for a total of eight injections	1	2.04	[122]
	1 mg/kg, injected on days 0, 2, 4, and 6	1	2.04	[118]
	1 mg/kg, injected on days 1, 3, 5, and 7	1	2.04	[117]
	2 mg/kg, injected on days 0, 2, 4, and 6	9	18.37	[91,94,96,98,102,106,109,110,120]
	2 mg/kg, injected on days 1, 3, 5, and 7	3	6.12	[26,99,112]
	2 mg/kg, injected on days 1, 3, 5, and 8	1	2.04	[125]
	2 mg/kg, injected on days 1, 4, 7, and 11	1	2.04	[107]
	4 mg/kg, injected on days 0, 2, 4, and 6	3	6.12	[110,103,105]
	4 mg/kg, injected on days 1, 3, 5, and 7	2	6.12	[104,121]
	8 mg/kg, injected on days 1, 3, 5, and 7	2	4.08	[101,123]
	20 mg/kg, injected on days 15, 17, 19, and 21	1	2.04	[92]
Continuous injection protocols	1 mg/kg, injection on days 1–4	1	2.04	[95]
	1, 3, 6 mg/kg, injection on days 1–7	1	2.04	[90]
	2 mg/kg, injection on days 1–5	12	24.49	[88,89,111,113,114,115,116,124,130,131,132,133]
	4 mg/kg, injection on days 1–4	1	2.04	[128]
	4 mg/kg, injection on days 1–5	2	4.08	[119,135]
	4 mg/kg, injection on days 1–8	1	2.04	[108]
Single administration protocols	6 mg/kg, injected once	2	4.08	[97,109]
	8 mg/kg, injected once	2	4.08	[126,127]
	Total	49	100	

### 3.5 Modeling safety

Of the 128 reviewed publications, 33 explicitly reported an absence of fatalities in both rats and mice. A single study documented two deaths during PIPNP model induction using a regimen of 16 mg/kg administered weekly over 5 weeks; one rat died following the initial dose, and another after the fourth injection [75]. The remaining 94 publications did not mention mortality outcomes. The specific data are summarized in Table 9.

**Table 9:** PIPNP model – modeling safety and their usage frequency

Safety	Frequency (instances)	Frequency/total (%)	Refs.
Clearly document that no animal deaths occurred	33	25.78	[13,15,19,21,22,29,30,36,38,39,43,45,46, 51,53,58,61,62,64, 68,70,80, 87,91,96,98,100,103,110,112,117,120]
Clearly record the number of deaths	1	0.78	[75]
Deathless description	94	73.44	[9,10,11,12,14,16,17,18,20,23, 24,25,26,27,28,31,32,33,34, 35,37,40,41,42,44,47,48,49,50,42,54, 55,56,57, 59,60,63,65,66,67,69,71,72, 73,74,76,77,78,79,81,82,83,84,85,86, 88,89,90,92,93,94,95,97,101,102,104,105, 106,107,108,109,111,113,114,115,116,118,119, 121, 122,123,124,125,126,127,128,129,130,131,132,133,134,135,136]
Total	128	100	

### 3.6 Pain-related behaviors in established model

PIPNP in clinical populations typically presents as numbness, tingling, pain, and sensory disturbances in the extremities. Comparable behavioral phenotypes have been observed in rodent models, mirroring the sensory abnormalities reported in humans. Based on established interspecies dose-conversion formulas and pharmacokinetic differences, a single paclitaxel dose of 1.0–32.0 mg/kg and cumulative exposure of 8.0–82.0 mg/kg in humans correspond to 1.3–12.9 mg/kg in rats. Similarly, human regimens involving single doses of 4.0–18.0 mg/kg and cumulative doses of 4.0–38.0 mg/kg translate to 0.33–3.1 mg/kg in mice. Within these dosage ranges, both rats and mice consistently exhibit characteristic behavioral alterations following paclitaxel administration, initially including pallor of distal limbs, elevated toe-licking frequency, and increased paw-lifting episodes. As nociceptive alterations progress, hallmark signs such as mechanical allodynia and thermal hypersensitivity emerge [139,140]. In the development of rodent PIPNP models, mechanical allodynia (111 instances, 48.47%) and thermal hyperalgesia (63 instances, 27.51%) represented the most frequently reported and widely adopted behavioral endpoints for model validation. A total of 229 pain-related behavioral responses were documented, with individual studies often reporting multiple endpoints. Refer to Table 10 for detailed quantitative data.

**Table 10:** PIPNP model – pain behaviors and their usage frequency

Pain-related behaviors	Frequency (instances)	Frequency/total (%)	Refs.
+MA	111	48.47	[9,10,11,12,13,14,15,16,17,18,19,20,21,22,23, 24,25,26,27,28,29,30,31,34,35,36,37,38,39,40, 41,42,43,44,45,46,47,48,49,50,51,52,53,54,55, 56,57,58,59,61,62,63,64,65,66,67,68,69,70,71, 73,74,75,76,77,78,79,80,85,87,88,89, 90,91,93,100,103,114,116,117,118,119,120,122,123,124,126, 129,130,131,132,133,134,135,136]
+MH	18	7.86	[31,32,34,35,42,46,60,75,76,81,84, 86,92,101,121,123,127,128]
+CA	27	11.79	[12,31,38,41,56,77,78,82,83,86,88,89,91,92,94, 96,98,100,102,103,106,112, 118,124,130,133,135]
+CH	5	2.18	[9,44,73,120,122]
+TA	5	2.18	[33,75,83,111,115]
+TH	63	27.51	[11,12,18,20,21,23,24,26,29,30,31, 33,37,38,39,43,45,47,48,52,56,57,58, 60,62,65,68,71,75,77,78,82,90,92,93, 97,99,101,104,108,109,111,112,113,114,115, 118, 119,120,122,123,124,126,127,129,130,132,133,134,135]
Total	229	100	

Note: MA: Mechanical allodynia, MH: Mechanical hyperalgesia, CA: Cold allodynia, CH: Cold hyperalgesia, TA: Thermal allodynia, TH: Thermal hyperalgesia. Symbols: +, This behavior was assessed as pain-like following paclitaxel administration.

### 3.7 Assessment techniques for established model

Evaluation of model validity and drug efficacy relies on specific detection methods designed to quantify alterations in pain-related behaviors. Analysis of 128 publications identified 17 distinct validation techniques, cumulatively applied 238 times, as individual studies often incorporated multiple assessments. Among methods targeting mechanical allodynia and hyperalgesia, the Von Frey test emerged as the most prevalent, accounting for 103 instances (43.28%), followed by the Randall-Selitto paw pressure test with 12 instances (5.04%). Cold sensitivity was evaluated using four techniques, with the acetone test being the most frequently employed (26 instances, 26%). Assessment of thermal sensitivity involved five methodologies, with the plantar test – applying thermal stimuli to the hind paw – being the most widely adopted (41 instances, 16.82%). Comprehensive data are summarized in Table 11.

**Table 11:** PIPNP model – assessment techniques and their usage frequency

Pain-related behaviors	Assessment techniques	Frequency (instances)	Frequency/total (%)	Refs.
+MA, +MH	Von Frey test	103	43.28	[9–21,23–27,29–32,34–60,63–66,69–71,73,78,81,84,85,88,90,91, 93,101,103, 104,106,109,111,112,117–124,126–129,131–136]
	Plantar dynamic test	9	3.78	[22,28,67,89,92,110,113,114,116]
	Mechanical claw pressure test	3	1.26	[61,87,105]
	Randall-Selitto foot pressure test	12	5.04	[34,35,42,46,62,68,75, 76,79,80,101,123]
	Pin prick test	2	0.84	[86,130]
	Tail pinch test	1	0.42	[130]
	Dynamic brush test	1	0.42	[31]
+CA, +CH	Acetone test	26	10.92	[9,12,38,41,44,73,78,82,86,88,89,91,94, 96,98,100,102,103,106, 112,118,120,124,130,133,135]
	Cold plate test	4	1.68	[56,111,122,125]
	Cold water bath and tail withdrawal method	5	2.10	[12,38,82,83,92]
	Cold water soaking test	1	0.42	[77]
+TA, +TH	Hot plate test	21	8.82	[12,20,24,30,31,33,37,38,48,58, 78,90,101,111,115,127,132,133]
	Thermal radiation rat rear foot method	40	16.81	[11,18,21,23,29, 31,39,43,45,47, 52,56,57,60,61,62,65,68,71,75,92,93, 97,99,101,104, 108,109,118,119,122, 124,126,129,130,134,135]
	Thermal radiation rat tail method	4	1.68	[72,77,82,130]
	Hot water bath and tail withdrawal method	5	2.10	[12,26,38,75,120]
	Hot-tail test	1	0.42	[83]
	Total	238	100	

### 3.8 Applications of the PIPNP model

Among the 128 reviewed studies, 96 (75%) established PIPNP models in rats or mice primarily for pharmacodynamic evaluation and mechanistic investigations. Of these, 27 studies (21.09%) concentrated on pharmacodynamic assessments, while 4 (3.13%) explored the underlying pathogenesis of PIPNP using animal models. A single study (0.78%) focused on characterizing behavioral phenotypes associated with the condition. Comprehensive data are provided in Table 12.

**Table 12:** PIPNP model – applications and their usage frequency

Applications	Frequency (instances)	Frequency/total (%)	Refs.
Pharmacodynamic evaluation + mechanistic investigations	96	75.00	[9,10,11,12,13,14,15,17,18,20,21,22,23,24,25, 26,28,29,30,31,32,34,35,36, 37,38,39,40,42,44,45,46,47,48,49,50, 52,53,54,55,56,58,59,60,61,62,63,64,65,66,67, 68,69,71,74,76,77,78,84,85,86,87,88,89,90,91, 92,95,97,98,99,100,101,102,104,106,108,109, 110,112,114,115,118,119,120,121,122,125,126,127,128,129,130,132,134,135]
Pharmacodynamic evaluation	27	21.09	[16,19,27,33,41,51,70,72,73,79,94, 96,103,105,107,111,113,116,117,123,124,131,133]
Underlying pathogenesis	4	3.13	[43,57,93,136]
Characterizing behavioral phenotypes	1	0.78	[75]
Total	128	100	

### 3.9 Experimental indicators

Current rat and mouse models of PIPNP were predominantly employed for pharmacodynamic evaluation, mechanistic exploration, and investigations into disease pathogenesis. Literature mining indicated that, depending on specific research aims, commonly examined tissues included the spinal cord, sciatic nerve, nerve root, and serum. Analytical methods such as Western blotting (WB), immunohistochemistry, and ELISA were frequently applied to quantify protein and mRNA expression levels associated with PIPNP. These molecular indicators supported both the interpretation of drug mechanisms and the investigation of PIPNP pathophysiology. For example, McCormick B’s study [51] demonstrated that MitoVitE attenuated paclitaxel-induced oxidative stress and mitochondrial dysfunction while reducing mechanical hyperalgesia in a rat model, implicating oxidative and mitochondrial disturbances in the condition’s etiology. The validity of the animal model was thereby reinforced by paclitaxel’s capacity to replicate these pathological features. To elucidate the methodological basis for model establishment, key experimental indicators and their usage frequencies are extracted from 128 studies and summarized in Table 13.

**Table 13:** PIPNP model – experimental indicators and their usage frequency

Experimental indicators	Specific indicator	Frequency (instances)	Frequency/total (%)	Refs.
Expression of related proteins	TPPV1	11	3.38	[24,26,30,31,35,44,48,98,106,109,114]
	TPPV4	2	0.62	[44,48]
	Nrf2	3	0.92	[54,101,120]
	HIF-1α	4	1.23	[16,29,80,105]
	HO-1	4	1.23	[16,54,101,120]
	Iba1	17	5.23	[25,48,49,52,54,57,60,64,71,77,110,114,125,127,135,136]
	CREB	2	0.62	[44,122]
	GFAP	30	9.23	[22,29,32,37,40,42,45,47,48,49,50,52,53,54,55,57, 60,65,71,76,77, 84,110,115,121,125,126,132,135,136]
	NLRP3	2	0.62	[66,101]
	ERK1/2	4	1.23	[46,88,92,99]
	P-ERK1/2	5	1.54	[32,46,88,92,99]
	NF-κB	4	1.23	[57,71,92,127]
	NF-κB P65	5	1.54	[22,40,46,78,90]
	P-NF-κB	1	0.31	[127]
	P-P38	3	0.92	[32,46,101]
	MAPK	2	0.62	[46,101]
	IB4	4	1.23	[44,47,50,127]
	P-AKT	4	1.23	[18,55,85,101]
	DNMT3a	3	0.92	[49,122,129]
	CGRP	5	1.54	[12,44,50,126,127]
	NeuN	5	1.54	[28,49,50,52,54]
	NGF	1	0.31	[62]
	NKCC1	1	0.31	[64]
	GLT-1	4	1.23	[10,29,32,42]
	TLR4	2	0.62	[22,95]
	iNOS	1	0.31	[127]
	PI3K	2	0.62	[55,101]
	c-FOS	4	1.23	[28,108,110,127]
	c-Jun	2	0.62	[90,122]
	P-JNK	2	0.62	[32,90]
	PGC-1α	1	0.31	[17]
	RIP3	1	0.31	[15]
	MAP2	2	0.62	[28,45]
	CD68	4	1.23	[37,77,85,120]
	CD11b	3	0.92	[84,114,132]
	OX-42	3	0.92	[42,45,50]
	PGC-1α	3	0.92	[17,18,39]
Expression of related mRNA	OX-42	3	0.92	[42,45,50]
	Nav1.7	2	0.62	[44,50]
	K2p1.1	2	0.62	[56,128]
	TNF-α	29	8.92	[12,15,21,22,23,37,40,42, 44,48,55,57,59,60,65,71,78,84,90, 92,95,97,99,104,120,123,134]
	IL-1β	26	8.00	[12,14,15,18,21,22,23,29,37,38, 40,46,48,55,57,59,60,61,66,78, 79,92,95,120,123,134]
	IL-6	15	4.62	[12,21,37,40,44,48,57,60,61,84, 92,99,104,123,134]
	IL-4	4	1.23	[42,47,61,120]
	IL-10	11	3.38	[18,23,42,46,47,57,61,84,99,120,121]
	MDA	6	1.85	[18,38,39,66,79,130]
	SOD	7	2.15	[18,21,39,42,66,86,114]
	GSH	4	1.23	[38,79,86,130]
	ROS	2	0.62	[66,120]
	Acetylated histone H4	2	0.62	[40,52]
	Acetylated histone H3	4	1.23	[52,56,122,129]
	Mitochondrial membrane potential (MMP)	1	0.31	[49]
	TRPV1	3	0.92	[44,98,114]
	Iba1	1	0.31	[52]
	NEuN	1	0.31	[28]
	GFAP	2	0.62	[84,115]
	c-FOS	2	0.62	[28,44]
	TLR4	1	0.31	[57]
	NF-κB p65	1	0.31	[57]
	DNMT3a	1	0.31	[49]
	c-Jun	1	0.31	[44]
	Nav1.7	2	0.62	[44,50]
	GLT-1	1	0.31	[115]
	GLuA1	1	0.31	[115]
	PGC-1α	1	0.31	[39]
	Nrf2	1	0.31	[114]
	CCL2	1	0.31	[84]
	CD11b	1	0.31	[84]
	KCNQ5	1	0.31	[136]
	TNF-α	6	1.85	[12,21,38,84,123,134]
	IL-1β	6	1.85	[12,38,44,120,123,134]
	IL-6	6	1.85	[12,38,44,84,123,134]
	IL-4	1	0.31	[120]
	IL-10	3	0.92	[47,84,120]
Cytological examination	Transmission electron microscopy (TEM) was employed to examine mitochondrial morphology	1	0.31	[89]
	Ultrastructural alterations in the sciatic nerve were visualized using TEM	2	0.62	[67,68]
	Toluidine blue staining was utilized to evaluate axonal degeneration	1	0.31	[74]
Plasma biochemical indexes	NO (nitric oxide)	2	0.62	[38,39]
	5-HT	1	0.31	[63]
	Total	325	100	

## 4 Evaluation and application analysis of the PIPNP animal model

The pathogenesis of PIPNP remains poorly understood. Current evidence indicates a multifactorial origin involving chemotherapy-induced mitochondrial impairment, oxidative stress, dysregulation of ion channel activity, neuroimmune dysfunction, dorsal root ganglion sensory neuron injury, and microvascular alterations [141]. This limited mechanistic insight continues to constrain the development of effective preventive and therapeutic interventions. Establishing a reproducible and reliable PIPNP animal model necessitates rigorous parameter optimization, including selection of animal strain, sex, developmental stage, paclitaxel dosage, modeling protocol, and outcome measures.

### 4.1 Evaluation and application analysis of animal species

The bibliometric analysis reveals that the rodent models are exclusively employed in current PIPNP model establishment, primarily due to their cost-effectiveness in husbandry, rapid reproductive cycle, and accelerated growth characteristics. Among rat models, SD rats predominate (68 instances, 51.91%), demonstrating enhanced pharmacological sensitivity compared to Wistar rats, which may potentiate intervention efficacy [142,143,144]. In mouse models, C57BL/6J and CD1 strains are frequently utilized (8 instances, 6.11%). However, CD1 mice as closed-colony stocks exhibit genetic heterogeneity and substantial phenotypic variability, potentially compromising experimental reproducibility [145]. In contrast, C57BL/6J – the inaugural mouse strain with fully sequenced genome – possesses a homogeneous genetic background that minimizes experimental variability [146]. Moreover, their immune system shares notable homology with humans, rendering this strain particularly suitable for mechanistic investigations into disease pathogenesis and pharmacological actions [147]. These comparative advantages strongly support the preferential selection of SD rats or C57BL/6J mice for optimized PIPNP model development.

### 4.2 Evaluation and application analysis of animal age

The 128 studies included 11 developmental stages, with animal ages ranging from 5 weeks to 10 months in both rats and mice. The most commonly selected age intervals were 6–8 weeks (17 instances, 13.28%) and 8–12 weeks (23 instances, 17.97%). Evidence suggests that early-life stressors, including maternal separation, may predispose young rats to depressive phenotypes in adulthood [148]. Accordingly, experimental modeling should utilize sexually mature adult rodents, with established maturation thresholds at 8–10 weeks for SD rats and 6–8 weeks for C57BL/6J mice, to avoid developmental variability associated with juvenile specimens.

### 4.3 Evaluation and application analysis of animal gender

Analysis of the literature revealed that male rodents were predominantly used for model induction, accounting for 96 sessions (75%). Although human studies have demonstrated greater experimental pain sensitivity in females across various modalities [149], no significant differences in the incidence or severity of PIPNP have been identified between sexes in preclinical models [150], indicating comparable pain responses during PIPNP progression. Nonetheless, numerous studies have shown that estrogen influences multiple signaling pathways within the nervous system. It alleviates neuropathic pain by suppressing microglial and astrocytic activation and downregulating pro-inflammatory cytokines such as IL-1β and IL-6 [151]. Additionally, estrogen enhances inhibitory neurotransmission via upregulation of NMDA receptor 1 and provides neuroprotection by mitigating oxidative neuronal damage [152,153]. Through its regulatory effects on neuroinflammation, neuronal excitability, and oxidative stress, estrogen exerts a multifactorial role in neuropathic pain modulation. Given these neuroregulatory functions, estrogen may confound the evaluation of paclitaxel-induced neurotoxicity when female rodents are employed in PIPNP models. Although direct sex-based comparative data in PIPNP modeling are limited, the established influence of estrogen on the peripheral nervous system supports the continued use of male rodents to minimize hormonal interference and improve model consistency.

### 4.4 Evaluation and application analysis of paclitaxel solvent

Literature mining identified Cremophor EL/ethanol (1:1) combined with 0.9% saline as the most frequently utilized solvent system for paclitaxel (38 instances, 29.69%), followed by direct dilution with 0.9% saline (24 instances, 18.75%). The choice of solvent largely depends on the formulation of paclitaxel. Owing to its poor aqueous solubility, conventional paclitaxel injections often require solubilizing excipients such as polyoxyethylene castor oil and ethanol. In preclinical models, alternative agents like DMSO and Tween 80 are also employed, though their inherent toxicity necessitates stringent control over concentration and dosing. Albumin-bound paclitaxel, formulated with human albumin as a carrier and stabilizer, exhibits high aqueous solubility and typically requires only dilution with 0.9% saline prior to administration [154,155,156]. Solvent selection should therefore align with the physicochemical properties of the paclitaxel formulation applied in each study.

### 4.5 Evaluation and application analysis of modeling protocols

Comprehensive analysis of the literature identified three primary paclitaxel-based modeling approaches: continuous injection, alternate-day injection, and single administration. All were validated as effective through various behavioral assessments. Among these, the 2 mg/kg dosing regimen administered on days 1, 3, 5, and 7 is recommended for both rats and mice, as it demonstrates superior alignment with clinical PIPNP symptomatology. This protocol most accurately replicates the characteristic phenotypes observed in PIPNP patients, who commonly present with distal limb numbness, tingling, and exaggerated responses to normally non-noxious stimuli. In rodent models, these clinical manifestations are paralleled by measurable reductions in mechanical pain thresholds and increased nociceptive sensitivity – key behavioral endpoints used to quantify symptom severity [157]. Data mining results indicate that 111 of 128 studies reported altered mechanical pain thresholds, with 25 of these employing the 2 mg/kg, days 1, 3, 5, and 7 protocol – the highest incidence among all dosing regimens. Similarly, among 63 studies documenting thermal hyperalgesia, 15 utilized this same schedule, again representing the highest proportion. Comprehensive data are provided in Table 14. These findings highlight a strong correlation between this dosing strategy and the induction of pain behaviors most representative of the PIPNP clinical symptomatology. This may be related to the prolonged half-life of paclitaxel in the body, as alternate-day injections can lead to repeated accumulation of the drug in the nervous system, preventing complete metabolism and resulting in prolonged exposure of nerves to toxic environments, thereby enhancing neuropathological responses [158,159,160]. Accordingly, we support its preferential adoption in preclinical modeling. From a pathological perspective, although the mechanisms underlying PIPNP remain unresolved, clinical trials have reported that certain patients undergoing paclitaxel chemotherapy exhibit not only neuropathic pain but also elevated serum IL-6 and IL-10 levels [161,162,163,164]. Data mining of 128 studies revealed that IL-6 levels were assessed in 16 cases, with 3 employing the 2 mg/kg dosing protocol on days 1, 3, 5, and 7 – representing the highest frequency among all regimens. Similarly, among the 11 studies measuring IL-10, this protocol appeared in 3 instances, again constituting a higher proportion. Comprehensive data are provided in Table 15. These patterns suggest a closer alignment between the 2 mg/kg, days 1, 3, 5, and 7 regimen and the inflammatory biomarkers observed in clinical PIPNP. Additionally, this protocol reflects the clinical pharmacological profile more accurately than single or continuous injections. The standard paclitaxel monotherapy regimen involves 175 mg/m^2^ administered intravenously over 3 h every 3 weeks [165]. The alternate-day dosing schedule better mirrors this clinical cadence. Furthermore, the 2 mg/kg dosage corresponds to the human equivalent dose derived from established interspecies conversion formulas, ensuring both translational relevance and safety in preclinical settings. Overall, the 2 mg/kg injection administered on days 1, 3, 5, and 7 establishes an animal model that better replicates the clinical and pathological attributes of PIPNP, offering a more representative platform for mechanistic investigation and therapeutic development.

**Table 14:** Modeling protocols for mechanical allodynia and thermal hyperalgesia: Frequency analysis

Pain-related behavior	Modeling protocols	Frequency (instances)	Frequency/total (%)	Refs.
+MA	2 mg/kg, injected once every other day, for a total of four injections	4	3.60	[28,36,51,134]
	2 mg/kg, injected every 4 days for a total of four injections	1	0.90	[76]
	3 mg/kg, injected once every other day, for a total of four injections	1	0.90	[70]
	4 mg/kg, injected once every other day, for a total of four injections	1	0.90	[129]
	4 mg/kg, injected once every other day, for a total of six injections	1	0.90	[122]
	4 mg/kg, injected once every other day, for a total of eight injections	1	0.90	[93]
	6 mg/kg, injected once a week for 4 weeks	1	0.90	[74]
	16 mg/kg, injected once a week for 5 weeks	1	0.90	[72]
	1 mg/kg, injected on days 1, 3, 5, and 7	4	3.60	[75]
	1 mg/kg, injected on days 0, 2, 4, and 6	2	1.80	[41,118]
	2 mg/kg, injected on days 0, 2, 4, and 6	24	21.62	[11,15,17,19,20,22,23,30,42,46, 53,54,64, 80,87,91,94,96,98,106,109,110,120]
	2 mg/kg, injected on days 1, 3, 5, 7, and 9	1	0.90	[10]
	2 mg/kg, injected on days 1, 3, 5, and 7	25	22.52	[13,14,18,24,26,29,31,34,35,43,45,48,50, 58,59,61,62,66,67,68,78,79,99,112,136]
	2 mg/kg, injected on days 1, 4, 7, and 11	1	0.90	[107]
	2.47 mg/kg, injected on days 1, 3, 5, and 7	2	1.80	[65,71]
	3, 6 mg/kg, injected on days 1, 2, 8, 9, 15, 16, 22, and 23	1	0.90	[9]
	4 mg/kg, injected on days 1, 4, 8, and 11	1	0.90	[73]
	4 mg/kg, injected on days 0, 2, 4, and 6	4	3.60	[56,100,103,105]
	4 mg/kg, injected on days 1, 3, 5, and 7	3	2.70	[12,38,104]
	4, 6 mg/kg, injected on days 1, 8, and 15	1	0.90	[57]
	5 mg/kg, injected on days 0, 2, 4, and 6	1	0.90	[69]
	5 mg/kg, injected on days 0, 7, 14, and 21	1	0.90	[27]
	8 mg/kg, injected on days 1, 4, and 7	5	4.50	[40,47,49,52,55]
	8 mg/kg, injected on days 0, 3, and 6	1	0.90	[85]
	8 mg/kg, injected on days 1, 3, 5, and 7	1	0.90	[125]
	8 mg/kg, injected on days 1, 3, 5, 7, and 9	1	0.90	[25]
	9 mg/kg, injected on days 0, 2, 4, and 6	1	0.90	[77]
	1 mg/kg, injected on days 1–4	1	0.90	[95]
	1, 3, 6 mg/kg, injected on days 1–7	1	0.90	[90]
	2 mg/kg, injected on days 1–4	1	0.90	[44]
	2 mg/kg, injected on days 1–5	11	9.91	[86,89,111,113,114, 116,124,130,131,132,133]
	4 mg/kg, injected on days 1–5	1	0.90	[119,135]
	4 mg/kg, injected on days 1–8	2	1.80	[108]
	6 mg/kg, injected once	1	0.90	[109]
	8 mg/kg, injected once	1	0.90	[126]
	5, 10 mg/kg, injected once	1	0.90	[37]
Total		111	100	
+TH	2 mg/kg, injected once every other day, for a total of four injections	1	1.59	[134]
	3 mg/kg, injected once every other day, for a total of four injections	1	1.59	[70]
	4 mg/kg, injected once every other day, for a total of four injections	1	1.59	[129]
	4 mg/kg, injected once every other day, for a total of six injections	1	1.59	[122]
	4 mg/kg, injected once every other day, for a total of eight injections	1	1.59	[93]
	16 mg/kg, injected once a week for 5 weeks	1	1.59	[75]
	1 mg/kg, injected on days 0, 2, 4, and 6	1	1.59	[118]
	1 mg/kg, injected on days 1, 3, 5, and 7	2	3.17	[21,39]
	2.47 mg/kg, injected on days 1, 3, 5, and 7	3	4.76	[60,65,71]
	2 mg/kg, injected on days 0, 2, 4, and 6	6	9.52	[11,20,23,30,109,120]
	2 mg/kg, injected on days 1, 3, 5, and 7	15	23.81	[18,24,26,29,31,43,45, 48,58,61,62,68, 78,99,112]
	4 mg/kg, injected on days 0, 2, 4, and 6	1	1.59	[56]
	4 mg/kg, injected on days 1, 3, 5, and 7	3	4.76	[12,38,104]
	4, 6 mg/kg, injected on days 1, 8, and 15	1	1.59	[57]
	5 mg/kg, injected on days 0, 2, 4, and 6	1	1.59	[69]
	8 mg/kg, injected on days 1, 4, and 7	2	3.17	[47,52]
	8 mg/kg, injected on days 1, 3, 5, and 7	2	3.17	[101,123]
	9 mg/kg, injected on days 0, 2, 4, and 6	1	1.59	[77]
	20 mg/kg, injected on days 15, 17, 19, and 21	1	1.59	[92]
	1, 3, 6 mg/kg, injected on days 1–7	1	1.59	[90]
	2 mg/kg, injected on days 0–6	1	1.59	[82]
	2 mg/kg, injected on days 1–5	9	14.29	[33,111,113,114,115,124,130,132,133]
	4 mg/kg, injected on days 1–5	1	1.59	[119,135]
	4 mg/kg, injected on days 1–8	2	3.17	[108]
	5, 10 mg/kg, injected once	1	1.59	[37]
	6 mg/kg, injected once	1	1.59	[107]
	8 mg/kg, injected once	2	3.17	[126,127]
Total		63	100	

**Table 15:** Modeling protocols for IL-6 and IL-10 detection: Frequency analysis

Experimental indicators	Modeling protocols	Frequency (instances)	Frequency/total (%)	Refs.
IL6	2 mg/kg, injected once every other day, for a total of four injections	1	6.25	[134]
	2 mg/kg, injected every 5 days, for a total of five injections	1	6.25	[84]
	1 mg/kg, injected on days 1, 3, 5, and 7	1	6.25	[21]
	2.47 mg/kg, injected on days 1, 3, 5, and 7	1	6.25	[60]
	2 mg/kg, injected on days 1, 3, 5, and 7	3	18.75	[48,61,99]
	4 mg/kg, injected on days 1, 3, 5, and 7	3	18.75	[12,38,104]
	4, 6 mg/kg, injected on days 1, 8, and 15	1	6.25	[57]
	8 mg/kg, injected on days 1, 3, 5, and 7	1	6.25	[123]
	8 mg/kg, injected on days 1, 4, and 7	1	6.25	[40]
	20 mg/kg, injected on days 15, 17, 19, and 21	1	6.25	[92]
	2 mg/kg, injected on days 1–4	1	6.25	[44]
	5, 10 mg/kg, injected once	1	6.25	[37]
Total		16	100	
IL10	2 mg/kg, injected every 5 days, for a total of five injections	1	9.09	[84]
	2 mg/kg, injected on days 0, 2, 4, and 6	4	36.36	[23,42,46,120]
	2 mg/kg, injected on days 1, 3, 5, and 7	3	27.27	[18,61,99]
	2 mg/kg, injected on days 1, 3, 5, and 7	1	9.09	[121]
	4, 6 mg/kg, injected on days 1, 8, and 15	1	9.09	[57]
	8 mg/kg, injected on days 1, 4, and 7	1	9.09	[47]
Total		11	100	

### 4.6 Evaluation and application analysis of pain behavior and assessment techniques

Literature mining indicated that mechanical allodynia (111 instances, 48.47%) and thermal hyperalgesia (63 instances, 27.51%) were the most frequently evaluated behavioral endpoints for validating PIPNP animal models. Mechanical hypersensitivity is conventionally assessed by delivering incremental mechanical stimuli to determine the minimum force eliciting a withdrawal response. This approach provides a reliable method for pain quantification in non-verbal subjects, as it enables objective assessment and yields reproducible results. Furthermore, the presence of mechanical allodynia correlates with the severity and duration of neuropathic pain, serving as a predictor of patient prognosis and risk for chronic pain development [166,167]. The widespread adoption of the Von Frey filament test underscores its methodological robustness in detecting mechanical pain thresholds [168]. The Thermal Nociceptive Sensitivity Assay enables consistent evaluation of pain sensitivity in laboratory animals by applying controlled heat stimuli and determining the threshold temperature that elicits a nociceptive response. Similar to mechanical pain assessments, it is particularly appropriate for subjects incapable of self-reporting. In addition, thermal hyperalgesia demonstrates high sensitivity to alterations in pain perception thresholds, capable of detecting even subtle changes in nociceptive thresholds. This not only mirrors peripheral nociceptor sensitization but also provides insights into central nervous system pain modulation dynamics [169,170]. Among thermal-based methods, the hot plate test remains the benchmark for quantifying nociceptive sensitization in rodents, offering high reproducibility through standardized temperature settings and precise measurement of response latency. Compared to other methodologies such as tail flick, thermal radiation, or paw/incisor assays, the hot plate test inflicts minimal somatic trauma on animals while mitigating stress-related confounding variables. This ensures more reliable quantifications of pain sensitivity with enhanced experimental reproducibility and specificity [171,172,173]. Therefore, we propose the implementation of both hot plate and Von Frey testing to evaluate alterations in mechanical allodynia and thermal hyperalgesia during PIPNP animal model establishment, serving as critical criteria for validating model success.

### 4.7 Specific operation methods

#### 4.7.1 Von Frey test

Animals were individually housed in chambers and permitted a 30 min acclimatization period. Commencing with a 2 g filament, a standardized series of Von Frey filaments (1, 1.4, 2, 4, 6, 8, 10, and 15 g) were applied perpendicularly to the plantar mid-region of the hind paw, exerting sufficient force to induce filament bending. A positive response was defined as either abrupt paw withdrawal or licking behavior upon filament stimulation. The minimum force (in grams) required to elicit a positive response was recorded as the paw withdrawal threshold. Given paclitaxel’s induction of bilateral allodynia, threshold values from both hind paws were averaged to yield a final measure [9,11].

#### 4.7.2 Hot plate test

The subject was retrieved from its housing cage and positioned on the heated surface of the hot plate apparatus for a 10–15 min habituation period. During this phase, the plate remained at ambient temperature, and ambulatory activity was restricted to a cylindrical acrylic enclosure featuring a ventilated upper portion. Following environmental acclimatization, the hot plate was equilibrated to and sustained at 52.0°C ± 0.1°C. Thermal stimulation was initiated upon temperature stabilization, with response timing synchronized via a foot-operated switch integrated with the apparatus. Nociceptive behaviors, defined as hind paw elevation or directed licking, immediately terminated the trial, and corresponding latency measurements were documented. A safety cutoff of 30 s was enforced to mitigate thermal injury risk, in compliance with ethical guidelines for thermal nociception assays [12,20].

### 4.8 Analysis of experimental indicators

Paclitaxel has been shown to trigger neuroinflammation through the release of pro-inflammatory and chemotactic mediators, alongside the recruitment of non-resident macrophages into the spinal cord and dorsal root ganglia (DRG). This cascade promotes glial activation and accumulation, ultimately contributing to the development of neuropathic pain [174]. Literature mining highlights a higher frequency of reported expression for several proteins and mRNAs – namely, Iba1, GFAP, CD68, CD11b, CCL2, TNF-α, IL-1β, and IL-6 – compared to other molecular markers. Among them, TNF-α, IL-1β, and IL-6 are well-established pro-inflammatory cytokines implicated in neuropathic mechanisms [175]; CCL2, a chemokine with strong chemotactic activity, mediates a range of pro-inflammatory responses [176]; CD68 and CD11b are markers predominantly expressed by macrophages and mononuclear phagocytes; GFAP indicates astrocyte reactivity [177]; and Iba1 serves as a hallmark of microglial activation [178]. Inflammatory signaling pathways such as NF-κB, MAPK, and TLR4 are also frequently reported, suggesting a consistent mechanistic pattern across studies. Collectively, these observations support the association between successful PIPNP model establishment and paclitaxel-induced neuroimmune alterations. In parallel, accumulating evidence links chemotherapeutic agents to ion channel dysregulation in DRG neurons. Altered channel function leads to increased action potential amplitude and firing frequency, heightened neuronal excitability, and spontaneous ectopic discharges, all of which contribute to neuropathic pain pathogenesis [179]. Literature mining further identifies Nav1.7, a voltage-gated sodium channel primarily expressed in peripheral nociceptors, as a key modulator of nociceptive signal propagation [180]. These data converge on the conclusion that paclitaxel disrupts ion channel homeostasis, playing a significant role in model pathophysiology. Additionally, elevated reactive oxygen species (ROS) levels in DRG neurons have been implicated in the maintenance of hypersensitivity in paclitaxel-induced neuropathic pain states [181]. Furthermore, paclitaxel-induced mechanical hyperalgesia has been linked to spinal oxidative stress, which sustains central sensitization and contributes to nociceptive signaling. Excessive ROS production activates aberrant ion channel responses, leading to mitochondrial membrane depolarization, disruption of respiratory chain complexes, and release of pro-apoptotic proteins – cascading into mitochondrial dysfunction. This compromise in mitochondrial integrity disrupts intracellular signaling and interferes with physiological transmission pathways [182]. Literature mining identified MDA, SOD, ROS, GSH, and MMP as commonly employed biomarkers. Among them, MDA, SOD, ROS, and GSH serve as established indicators of oxidative stress, whereas MMP provides a quantitative measure of mitochondrial function. These data corroborate the mechanistic framework described above, indicating that the PIPNP model reflects paclitaxel-induced mitochondrial impairment and redox imbalance in DRG neurons. Moreover, chemotherapeutic agents have been reported to induce persistent NET accumulation, triggering microcirculatory impairment in peripheral tissues, including limbs, sciatic nerves, and DRGs, ultimately resulting in localized ischemia and hypoxia [141]. The frequent citation of HIF-1α across studies further supports its involvement in mediating cellular adaptation to hypoxic stress. Collectively, the literature-based analysis substantiates the role of paclitaxel-induced microvascular dysfunction as a core mechanism underlying PIPNP pathogenesis. Furthermore, research conducted by Li et al. identified TRPV1 as a key mediator in pain perception, with a prominent role in the progression of PIPNP [31]. Literature mining indicated consistent upregulation of TRPV1 at both the protein and mRNA levels, corroborating earlier mechanistic insights and linking paclitaxel administration to altered TRPV1 expression during model development. Beyond molecular profiling, TEM has been utilized to evaluate sensory neuron ultrastructure and detect cytological damage. This approach, highlighted in multiple studies, reinforces the association between paclitaxel exposure and structural neuronal injury, thereby supporting model validity. Collectively, the observed molecular and morphological alterations establish a mechanistic rationale for the effective construction of the PIPNP model.

**Figure 1:**
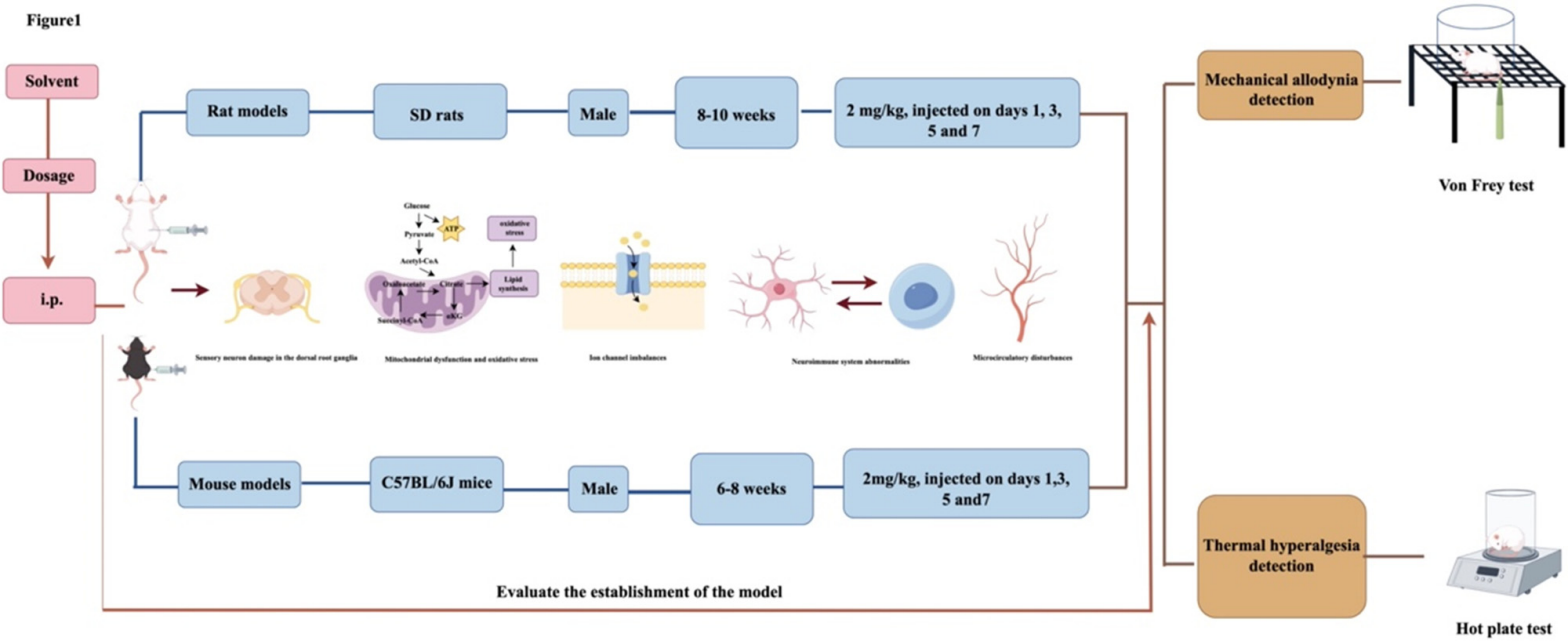
Flowchart for the development of a PIPNP animal model based on data mining outcomes and modeling evaluation analysis.

In summary, analysis of data mining outcomes and comparative evaluation of modeling protocols supports the use of 6- to 8-week-old male SD rats or 8- to 10-week-old male C57BL/6J mice as the preferred model organisms. Induction of the PIPNP model is achieved through intraperitoneal administration of paclitaxel at 2 mg/kg on days 1, 3, 5, and 7, a regimen that elicits mitochondrial dysfunction, oxidative stress, ion channel imbalance, neuroimmune alterations, sensory neuron damage within the DRG, and microcirculatory impairment. Mechanical allodynia and thermal hyperalgesia can be quantitatively assessed using the Von Frey and hot plate tests, respectively, to validate model establishment. The detailed experimental protocol is presented in Figure 1.

## 5 Discussion

The pathogenesis of PIPNP remains unclear, and selecting an appropriate animal model has become essential for advancing research in this area. An optimized PIPNP model not only supports the elucidation of underlying mechanisms but also serves as a foundational platform for drug screening and target validation, thereby enhancing the transition from preclinical pharmacodynamic studies to clinical applications. Moreover, effective models contribute to the refinement of therapeutic strategies and the development of preventive interventions. Rat and mouse PIPNP models provide distinct advantages, including methodological simplicity, reliable detection metrics, and broad applicability. These features have positioned rodent models as indispensable tools for investigating PIPNP-related neurobiological processes and evaluating emerging therapeutic approaches with translational potential.

In experimental research, aligning the selection of animal models with both the study objective and the specific attributes of each model is fundamental for ensuring data validity. A chronological review of the literature indicates that early PIPNP modeling efforts remained largely exploratory, primarily demonstrating mechanical hypersensitivity as the predominant phenotype. With the advancement of hypotheses regarding PIPNP pathogenesis, models have progressively incorporated additional pain modalities, including thermal and cold hypersensitivity, along with broader behavioral alterations. Contemporary model construction now involves multi-system, multi-dimensional evaluation strategies, accompanied by a gradual expansion in the scope of assessment parameters. Attention has increasingly shifted from purely behavioral endpoints toward integrative analyses including serological, cytological, and molecular dimensions. Technologies such as proteomics, cytomics, and genomics have become more widely implemented, reflecting a progression in research focus from organ-level observations to cellular and molecular interrogation. This methodological evolution has strengthened the robustness and translational relevance of criteria used to evaluate model fidelity.

In summary, current research on PIPNP animal models remains in a phase of refinement, with the primary challenge being the alignment between experimental models and clinical phenotypes. Enhancing this translational relevance requires addressing several limitations identified through literature mining. First, the implementation of a dynamic dose-adjustment strategy is warranted. Clinically, PIPNP represents a dose-dependent neurotoxic response, where symptom severity escalates with cumulative exposure [160]. However, most existing studies adopt fixed-dose regimens – typically involving alternate-day or continuous paclitaxel administration – without incorporating the adaptive dosing strategies common in clinical practice. While such approaches simulate general treatment patterns, they fail to capture the clinical course of PIPNP onset and progression. Future models should therefore integrate dose modulation, including reductions or temporary cessation upon the emergence of marked neurotoxicity. Monitoring the reversibility of symptoms following dose adjustment would further clarify the extent to which the model reflects the clinical dose-response relationship, thereby enhancing its predictive validity. Enhancing the alignment between PIPNP animal models and clinical phenotypes requires methodological refinement. Second, extending the observation window and optimizing the intervals for pain behavior assessments are essential. Recent evidence indicates that approximately 30% of paclitaxel-treated patients continue to experience PIPNP symptoms years after chemotherapy completion [44]. Correspondingly, preclinical studies have shown that pronounced nociceptive hypersensitivity in animals emerges around week 3 [92], with thermal hyperalgesia persisting beyond 8 weeks and mechanical allodynia extending up to 12 weeks [183]. Despite this, most current PIPNP models, both domestically and internationally, limit the modeling period to 21 days and the observation window to 56 days [89], resulting in behavioral assessments conducted almost exclusively within the first 3 weeks. Such temporal constraints may hinder accurate modeling of the chronic nature of clinical PIPNP. To improve translational relevance, future protocols should extend monitoring durations and refine the timing of behavioral evaluations accordingly. Third, a shift toward multimodal assessment strategies is warranted. Current model validation primarily relies on passively elicited responses, such as altered mechanical thresholds and evoked nociceptive hypersensitivity. However, clinical PIPNP is often characterized by spontaneous pain and sensory disturbances, including numbness – features not adequately captured by existing paradigms. This mismatch suggests that sole dependence on reflexive behavioral endpoints limits the model’s capacity to replicate the full clinical spectrum. Incorporating complementary assessment tools capable of detecting spontaneous pain behaviors may enhance model fidelity and provide a more comprehensive representation of clinical symptomatology. The nerve conduction velocity assay evaluates motor axonal function by stimulating the sciatic nerve and recording evoked muscle action potentials [184], while neuromuscular ultrasound enables real-time visualization of nerve swelling and muscle denervation [185]. These techniques provide dynamic, objective assessments of nociceptive alterations in PIPNP animal models, addressing the limitations inherent to behavioral testing. Future studies would benefit from a multimodal assessment framework that integrates behavioral, electrophysiological, and neuroimaging approaches to enhance model validity and better approximate the complexity of clinical pain. Fourth, the development of a PIPNP animal model aligned with both disease presentation and syndrome differentiation is essential. Literature mining on PIPNP mechanisms in traditional chinese medicine (TCM) indicates that existing models lack clear correspondence with clinical diagnostic patterns such as qi deficiency, blood deficiency, cold congealment, and blood stasis. Future investigations should prioritize constructing models that incorporate TCM pathophysiological classifications alongside established Western biomedical indicators. This integrative approach would strengthen the translational relevance of preclinical models by improving alignment with clinically observed phenotypes. While the present study primarily sourced literature from English databases, strategic incorporation of findings from Chinese repositories has established a methodological foundation for international utilization of PIPNP animal models. However, to facilitate global adoption of the optimized PIPNP model proposed herein, subsequent investigations should (1) conduct comparative analyses with established international models (e.g., spared nerve injury, chronic constriction injury) to validate superior alignment with human chronic pain pathophysiology and (2) develop alternative validation protocols for resource-constrained research settings, particularly through identification of molecular biomarkers potentially substituting traditional behavioral pain assessments.

Addressing the four aforementioned challenges demands continued methodological refinement to develop a robust animal model of PIPNP that more precisely recapitulates the pathophysiological complexity and clinical heterogeneity observed in human patients. A model with greater translational relevance would provide a robust framework for elucidating disease mechanisms and guiding the development of frontline therapeutic strategies aimed at effective prevention and symptom control, thereby enhancing patient quality of life.
